# Development of a Sildenafil Citrate Microemulsion-Loaded Hydrogel as a Potential System for Drug Delivery to the Penis and Its Cellular Metabolic Mechanism

**DOI:** 10.3390/pharmaceutics12111055

**Published:** 2020-11-04

**Authors:** Apichart Atipairin, Charisopon Chunhachaichana, Titpawan Nakpheng, Narumon Changsan, Teerapol Srichana, Somchai Sawatdee

**Affiliations:** 1 Drug and Cosmetics Excellence Center, Walailak University, Thasala, Nakhon Si Thammarat 80161, Thailand; apichart.at@wu.ac.th; 2 School of Pharmacy, Walailak University, Thasala, Nakhon Si Thammarat 80161, Thailand; 3 Drug Delivery System Excellence Center and Department of Pharmaceutical Technology, Faculty of Pharmaceutical Sciences, Prince of Songkla University, Hat Yai, Songkhla 90112, Thailand; ch.chari.21@gmail.com (C.C.); titpawan@gmail.com (T.N.); teerapol.s@psu.ac.th (T.S.); 4 College of Pharmacy, Rangsit University, Pathumtani 12000, Thailand; narumon.c@rsu.ac.th

**Keywords:** sildenafil citrate, microemulsion, hydrogel, skin permeation, stability, metabolism

## Abstract

Sildenafil citrate is used to treat mild to moderate erectile dysfunction and premature ejaculation. However, it has low oral bioavailability, numerous adverse effects, and delayed onset of action. These problems may be resolved by transdermal delivery to the penis. Hence, sildenafil citrate was formulated as a microemulsion system using isopropyl myristate, Tween 80, PEG400, and water (30:20:40:10). The hydrogel used in the microemulsion was 2% *w/w* poloxamer 188. The sildenafil microemulsion-loaded hydrogels were characterised for their appearance, particle size, pH, spreadability, swelling index, viscosity, sildenafil drug content, membrane permeation, epithelial cell cytotoxicity, and in vitro drug metabolism. The optimised formulated microemulsion showed the lowest droplet size and highest solubility of sildenafil citrate. The in vitro skin permeation of the sildenafil citrate microemulsion-loaded hydrogel was significantly higher than that of the sildenafil suspension, with a 1.97-fold enhancement ratio. The formulated microemulsion exhibited a 100% cell viability, indicating its safety for skin epithelial cells. The major metabolic pathway of sildenafil citrate loaded in the microemulsion formulation was hydroxylation. Furthermore, loading sildenafil in the microemulsion reduced the drug metabolite by approximately 50% compared to the sildenafil in aqueous suspension. The sildenafil citrate-loaded isopropyl myristate-based microemulsion hydrogels were physically and chemically stable over 6 months of storage. The sildenafil citrate microemulsion-loaded hydrogel showed in vitro results suitable for used as a transdermal drug delivery system.

## 1. Introduction

Sildenafil citrate is a selective phosphodiesterase type 5 (PDE5) inhibitor. The mechanism of action of sildenafil involves the specific degradation of cyclic guanosine monophosphate (cGMP), resulting in smooth muscle relaxation via the nitric oxide (NO) pathway [1]. Thus, sildenafil citrate is an effective oral treatment for erectile dysfunction and premature ejaculation [2,3]. It is available only in the form of tablets. Some studies have shown that sildenafil citrate or PDE inhibitor drugs can be administered topically [4,5,6,7]. The transdermal delivery of sildenafil citrate through a local tissue area could be considered as an alternative to the oral route, in order to avoid adverse effects, shorten the onset time, and sustain the therapeutic effects for longer periods [1]. The topical administration of sildenafil also has the advantage of the lack of significant systemic absorption, thus reducing systemic adverse effects. Therefore, delivery of the drug directly to the penis in a controlled manner would be preferable for men for whom oral medications are unsafe, or in whom this treatment could cause too many adverse effects. However, the lower efficacy of topical therapy compared to systemic therapy is a major limitation, especially in severe cases. Nonetheless, patients with mild and moderate erectile dysfunction and premature ejaculation may benefit from the accepted efficacy of topical therapy without systemic adverse effects. Results from randomised controlled trials on the efficacy of topical sildenafil in men with mild to moderate erectile dysfunction associated with premature ejaculation are limited and contradictory [2,6,8]. At present, two new treatment options, namely, topical anaesthetic agents and oral sildenafil, are being considered for treating premature ejaculation. Topical anaesthetic agents are applied to the glans penis before intercourse to delay ejaculation [2]; these agents do not show any dermal toxicity, and wound healing properties were noted against traumatic wounds [5]. The absorption of topical sildenafil depended on the ability of the drug to penetrate through the skin to the target receptor to reach therapeutic levels. The stratum corneum is the outermost epidermal layer consisting of keratin-filled dead cells called corneocytes. These cells hinder the penetration of drugs. This skin barrier is overcome by using the most widely implemented approach involving chemical penetration enhancers that can, ideally, safely and reversibly alter the physicochemical nature of the stratum corneum to facilitate drug delivery through the skin [4].

Microemulsions are attractive drug-delivery systems because of the following advantages: ease of preparation owing to the spontaneous formation, thermodynamic stability, transparent and elegant appearance, increased drug loading, enhanced penetration through the biological membranes, increased bioavailability, and less inter- and intra-individual variability in drug pharmacokinetics [9]. In addition, hydrogels contain a high water content and possess a degree of flexibility very similar to the natural tissue, are less greasy, and can be easily removed from the skin. Hydrogels have the ability to sense changes in pH, temperature, or metabolite concentration and release their load in response to such changes; they also possess good transport properties and can be modified easily [10].

**Figure 1:**
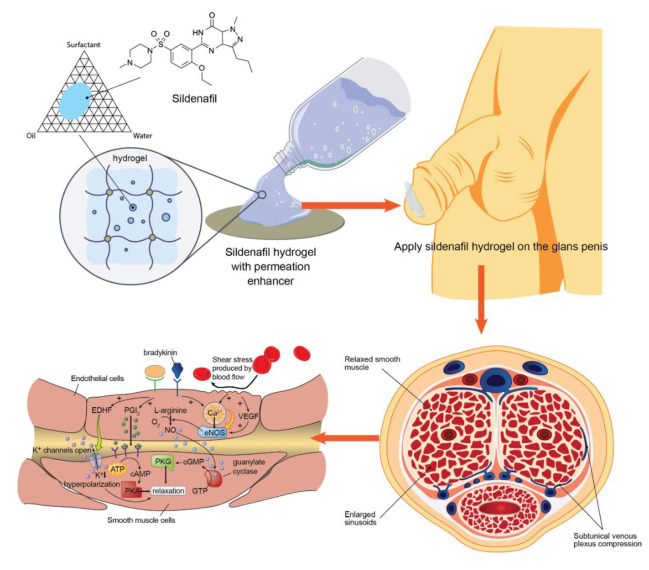
Schematic diagram of the sildenafil microemulsion-loaded hydrogels for transdermal drug delivery directly to the penis and smooth muscle cell relaxation mechanism (EDHF is endothelium derived hyperpolarizing factor; PGI_2_ is prostacyclin; NO is nitric oxide; eNOS is endothelial nitric oxide synthase; VEGF is vascular endothelial growth factor; ATP is adenosine triphosphate; GTP is guanine triphosphate; cAMP is cyclic adenosine monophosphate; cGMP is cyclic guanosine monophosphate; PKA is protein kinase A; PKG is protein kinase G).

In this study, sildenafil citrate transdermal delivery systems using microemulsion-loaded hydrogels were constructed and evaluated. The formulated microemulsion systems were characterised through visual inspection of their appearance, particle size, and pH and were analysed for spreadability, swelling index, cell membrane permeability, sildenafil drug content, and viscosity. Moreover, in vitro epithelial cell cytotoxicity and drug metabolism from the formulated systems was determined. The knowledge of the mechanism of action of the novel sildenafil citrate formulation may help in evaluating drug efficacy or be used to develop formulation systems. Finally, long-term and accelerated stability studies were performed under high-stress conditions. A schematic diagram of the entire research is shown in Figure 1.

## 2. Materials and Methods

### 2.1. Materials

Sildenafil citrate and its reference standard were purchased from Smilax Laboratories Limited (Hyderabad, India). Isopropyl myristate, oleic acid, olive oil, sesame oil, castor oil, polyethylene glycol 400 (PEG400), propylene glycol, Tween 80, Tween 40, Tween 20, Cremophore RH40, and Transcutol were procured from Namsiang Co., Ltd. (Bangkok, Thailand) and S. Tong Chemical Co., Ltd. (Nonthaburi, Thailand). Poloxamer 188 (Pluronic^®^ F-68) solution (10%) was purchased from BASF (Ludwigshafen, Germany). A Strat-M^®^ membrane was purchased from Merck Millipore (Darmstadt, Germany). All other chemicals were of USP or reagent grade and used without further purification.

### 2.2. Solubility Studies

Sildenafil citrate in excess amounts were mixed with 5 mL of various oils (isopropyl myristate, oleic acid, olive oil, sesame oil, and castor oil), surfactants (Tween 80, Tween 60, Tween 40, and Cremophore RH40), and co-surfactants (PEG400, propylene glycol, and Transcutol) along with distilled water in separate vials. The vials were tightly secured and shaken at 25 °C for 24 h in a thermostatically controlled shaking water bath (type 3047; Köttermann, Hänigsen, Germany). The suspensions were centrifuged at 3000× *g* at 37 °C in a Sigma centrifuge (Sigma-Aldrich GmbH, Osterode am Harz, Germany) for 10 min. The contents of each vial were filtered through a 0.45-μm nylon membrane filter, and the supernatant was assayed for drug content using high-performance liquid chromatography (HPLC), according to the method described in the following section.

### 2.3. Construction of Pseudo-Ternary Phase Diagram

This step was performed after a suitable oil, surfactant, and co-surfactant with good sildenafil citrate solubilization capacity were selected. The selected oils were isopropyl myristate and oleic acid, while the surfactants and co-surfactants were Tween 80, PEG400, and propylene glycol.

The region of microemulsion existence was determined by constructing the pseudo-ternary phase diagrams of the oil, surfactant/co-surfactant, and water using the water titration method at a temperature range of 25–30 °C. For each phase diagram at a specific ratio of surfactant/co-surfactant (1:1, 1:2, 1:3, 2:1, and 3:1), a transparent and homogenous mixture of surfactant and co-surfactant was formed by vortexing for 5 min. Mixtures of oil with the surfactant and co-surfactant were prepared at the ratios of 10:0, 9:1, 8:2, 7:3, 6:4, 5:5, 4:6, 3:7, 2:8, 1:9, and 0:10. Mixtures of oil and the surfactant/co-surfactant at certain mass ratios were then titrated with water and visually evaluated for transparency and flow properties by tilting the tube. The formation of the monophasic/biphasic system was confirmed by visual inspection. The endpoint of titration was the point where the mixture became turbid or phase separation was observed. If turbidity appeared before the phase separation, the samples were considered as biphasic. The samples were considered monophasic if clear and transparent mixtures were visualised after stirring. At that point, the amounts of water, oil, surfactant, and co-surfactant added were noted. Monophasic, clear, low-viscosity systems were considered as microemulsions and shown as the microemulsion region in the pseudo-ternary phase diagram [11,12].

### 2.4. Preparation of the Sildenafil Citrate Microemulsion-Loaded Hydrogel

The ratio of the composition of each component was selected according to the results of the microemulsion area on the pseudo-ternary phase diagram. The microemulsions prepared from the oil (isopropyl myristate or oleic acid), surfactant, and co-surfactant (Tween 80/PEG 400 and Tween 80/propylene glycol) in distilled water were prepared at room temperature. The solubilisation capacity of each microemulsion system for sildenafil citrate was investigated. Increasing amounts of sildenafil citrate were added portion-wise to 2 g of each microemulsion system with stirring until a clear and transparent liquid was obtained and the excess solid particles dissolved completely. The mixture was stirred at 25 °C for 24 h. The solubility of the sildenafil citrate was determined as described in Section 2.2, with filtration being performed without centrifugation. The clear sildenafil citrate dissolved in the microemulsion was determined by HPLC. From the results of the microemulsion selected from the pseudo-ternary phase diagram and the highest solubility of sildenafil citrate dissolved in the microemulsion system, the ratios of the microemulsion compositions were selected. The formulations and compositions of the sildenafil citrate microemulsion-loaded hydrogels are shown in Table 1.

In brief, 240 mg of sildenafil citrate was dissolved in 2 g of the selected microemulsion system and stirred until homogeneity was reached. Next, 500 mg of poloxamer 188 (10% solution) was dispersed into the sildenafil citrate microemulsion by stirring to form a homogeneous solution. Microemulsions without sildenafil citrate were added to 500 mg of poloxamer 188 and stirred until dissolution and used as the control. The final concentration of sildenafil citrate was 87.6 mg/g and that of poloxamer 188 was 2% *w/w*. The sildenafil citrate microemulsion-loaded hydrogels were stored in a tightly secured glass bottle at room temperature (25 ± 2 °C) and refrigerated (4 ± 2 °C) until further study [5,13].

### 2.5. Physical Properties of the Sildenafil Citrate Microemulsion-Loaded Hydrogel

#### 2.5.1. Physical Appearance and Clarity

The appearance and clarity of the sildenafil citrate dissolved in the microemulsion and the sildenafil citrate microemulsion-loaded hydrogels was visually inspected.

#### 2.5.2. Determination of Droplet Size

The average droplet size of the microemulsion samples was measured at 25 °C using a MalvernZetasizer (Worcestershire, UK) equipped with 200 Hydro MU. The microemulsion and sildenafil citrate microemulsion-loaded hydrogel (2.74 g) were transferred to a disposable polystyrene cuvette using a plastic syringe or micropipette, and the droplet size of the microemulsion was determined and calculated on the basis of the volume size distribution [14].

#### 2.5.3. pH Determination

The pH of the sildenafil citrate microemulsion-loaded hydrogel samples were determined, and were monitored using a digital pH meter (Model 704, Metrohm, Herisau, Switzerland). A suitable amount of hydrogel samples was taken in a beaker. The probe of the pH meter was immersed in the hydrogel, and the pH values were recorded. The pH was determined in triplicate, and the mean was recorded.

#### 2.5.4. Spreadability Test

The spreadability of the sildenafil citrate microemulsion-loaded hydrogel was determined according the method described by Osman [7]. Here, 0.5 g of each sample was placed on the centre of a glass slide within a pre-marked circle. Another glass slide was then placed over the previous one. Following that, 500 mg of a metallic weight mass was placed on top of the upper glass slide for 5 min. The spreadability was calculated as the increase in diameter from the initial diameter of the boundary of the sildenafil microemulsion-loaded hydrogel.

#### 2.5.5. Swelling Index

The extent of the swelling was measured in terms of the percentage weight gained by the hydrogel mass. For this, 1 g of each sildenafil citrate microemulsion-loaded hydrogel formulation was weighed and kept on a sieve placed on a petri dish containing 10 mL of buffer solution (pH 5.8). At the end of the specified time intervals, the sieve containing the hydrogels was withdrawn from the petri dish and the excess buffer was soaked with tissue paper and weighed. The percentage of weight gained by the hydrogel was calculated using Equation (1):

(1) $$\mathrm{Swelling}\;\mathrm{index}\;(\%)\;=\;\frac{M_{t}{\;-\;M}_{0}}{M_{0}}\times 100$$

where M_t_ is the weight of the sildenafil microemulsion-loaded hydrogel formulation at time t; and M_0_ is the initial weight of the hydrogel formulation [13].

### 2.6. Viscosity and Viscoelastic Measurements

The viscosity and viscoelasticity of the sildenafil citrate microemulsion-loaded hydrogel samples were analysed using a Modular Advanced Rheometer System (HAAKE MARS 60; ThermoFisher Scientific, Bremen, Germany). The equipment was fitted with a 60 mm diameter parallel plate configuration with a gap of 0.5 mm and a Peltier temperature control system. Samples were loaded onto the bottom plate, and the upper plate was then moved to the settled gap. The flow experiments were performed over a shear rate range of 1 to 1000 s^−1^ at a constant temperature of 25 °C. The temperature dependence of the viscoelasticity was determined using a temperature sweep from 5 to 40 °C at a heating rate of 1 °C/min at an oscillation frequency of 1 Hz. The time dependence of the viscoelasticity at the simulated physiological temperatures was evaluated using a time sweep at 1 Hz, while maintaining the temperature at 37 °C. A frequency sweep was performed within the frequency range of 0.1100 Hz at a constant temperature of 37 °C. All tests were performed in triplicate [15].

### 2.7. Fourier Transform Infrared Spectroscopy (FTIR)

A small amount of the sildenafil citrate powder and microemulsion-loaded hydrogel formulations (from both the isopropyl myristate-based and oleic acid-based systems) with and without sildenafil citrate were ground and mixed into KBr pellets using a small mortar and pestle. The sample with the KBr mixture paste was compressed into tablets using a hydraulic press before the measurement of the infrared (IR) spectrum at the ambient temperature. FTIR spectra were obtained using the PerkinElmer Spectrum One Spectrometer (Perkin Elmer Inc., Waltham, MA, USA). All spectra were collected at room temperature at a resolution of 2 cm^−1^, and the measurement range was 400–4000 cm^−1^. The data were processed using the spectrum software of PerkinElmer Spectrum One Spectrometer (Version 5.0.2).

### 2.8. Sildenafil Citrate Content in the Microemulsion-Loaded Hydrogel

The sildenafil citrate content in the microemulsion-loaded hydrogel was determined by HPLC according to the method published in previous studies [16,17]. The HPLC equipment (Ultimate 3000; Dionex Corporation, Sunnyvale, CA, USA) and the related system consisted of a solvent delivery pump, inline degasser, a sample loop with an injection volume of 20 μL, and an autosampler. The column is a reversed-phase, stainless-steel column (250 mm long × 4.6 mm internal diameter; Inertsil ODS-3, GL Sciences, Tokyo, Japan) filled with 5 μm octadecylsilane. The mobile phase consisted of a degassed mixture of 0.2 M ammonium acetate buffer and acetonitrile in a ratio of 40:60 by volume at an ambient temperature and adjusted to pH 7.0 with 0.1 N NaOH before use. The flow rate was maintained at 1.0 mL/min, and ultraviolet detection was performed at 240 nm. Data were recorded using Chromeleon 7 software. The method was validated for four parameters: specificity, linearity, precision, and accuracy. In addition, a system suitability parameter was calculated.

### 2.9. Stability Studies

Selected formulations of the sildenafil citrate microemulsion-loaded hydrogels (both of isopropyl myristate-based and oleic acid-based systems) were filled in screw-cap amber glass bottles (10 g) to block moisture and light. The samples were kept under accelerated conditions (40 °C/75% relative humidity (RH)) and long-term stability conditions (30 °C/75% RH), following the Association of Southeast Asian Nations Guidelines for studying the stability of drug products according to climatic zone IVb [18]. Both formulations were evaluated for appearance, sildenafil content, pH, and viscosity over 1, 3, and 6 months.

### 2.10. Cell Viability Assay

The human skin fibroblast cell line (BJ; ATCC^®^ CRL-2522™, Manassas, VA, USA) was cultured in Eagle’s Minimum Essential Medium (Gibco^®^, Billings, MT, USA) containing 10% foetal bovine serum (FBS; Gibco^®^, Grand Island, NY, USA) and antibiotics (100 U/mL penicillin and 100 U/mL streptomycin; Gibco^®^, Grand Island, NY, USA) under 5% CO_2_ at 37 °C. The cytotoxicity of sildenafil citrate microemulsion-loaded hydrogel formulation in the human skin fibroblast cell line was evaluated using the MTT method. The BJ cells were seeded into a 96-well plate at a concentration of 10^5^ cells/mL in Eagle’s Minimum Essential Medium containing 10% FBS. After 24 h, the sildenafil citrate microemulsion-loaded hydrogel formulation of various concentrations in fresh medium were added into the 96-well plates and untreated cells (without sample) served as the negative control. Subsequently, 3-(4,5-dimethylthiazolyl-2-yl)-diphenyl-tetrazolium bromide (MTT) solution (5 mg/mL) was used to evaluate cell activity. Briefly, after 24 h of incubation, the sample cells were treated with 50 µL of fresh media along with 50 µL of MTT solution and incubated at 37 °C under 5% CO_2_ for 4 h. Thereafter, media containing MTT were removed, and 100 µL of dimethyl sulfoxide was added to dissolve the formazan salt formed. The absorbance was determined using a microplate reader (Biohit 830, Biohit^®^, Helsinki, Finland) at a wavelength of 570 nm. The percentage of cell viability was calculated relative to the negative control. 

### 2.11. Skin Permeation Studies Using Franz Diffusion Cells

Transdermal permeation of sildenafil citrate from the microemulsion-loaded hydrogel formulations was studied and compared with transdermal permeation of the sildenafil citrate suspension (87.6 mg/mL of sildenafil citrate). The in vitro release and permeation of sildenafil citrate from selected sildenafil citrate microemulsion-loaded hydrogels were studied using an automated Franz diffusion cell apparatus (MicroettePlus; Hanson Research, Chatsworth, CA, USA). Strat-M^®^ membranes (Millipore, Billerica, MA, USA), a synthetic, non-animal-based model that can substitute for human skin during transdermal diffusion testing, were mounted between the donor and receptor chambers of the Franz diffusion cells, with an effective diffusion area of 1.76 cm^2^ and cell volume of 12 mL, filled with freshly prepared citrate buffer solution (pH 5.8). The receptor fluid consisted of a citrate buffer solution (pH 5.8). After an equilibration time of 15 min at 32 °C ± 0.5 °C, the sildenafil microemulsion-loaded hydrogel formulations (2 g) were applied to the donor compartment through direct contact with the Strat-M^®^ membranes. The diffusion cells were maintained in a shaking water bath model 3047 (Köttermann, Hänigsen, Germany). They were shaken at 32 °C ± 0.5 °C with 100 rpm. The samples (3 mL) were collected at 0.5, 1.5, 5, and 24 h at each point, and the fresh buffer was replaced in the cells. The withdrawn samples were analysed using a previously validated HPLC method. All experiments were performed in triplicate. The ability of both the developed sildenafil citrate microemulsion-loaded hydrogel formulations to enhance the flux of the drug through the Strat-M^®^ membranes was assessed and compared to the sildenafil citrate suspension at the same concentration.

Permeation parameters, including the steady-state transdermal flux *J*_ss_ (μg/cm^2^/h), permeability coefficient *P*_c_ (cm/h), and diffusion coefficients (D), were calculated from the in vitro membrane permeation data. The cumulative amount of sildenafil citrate permeated per unit area of the membrane was plotted as a function of time. The steady-state flux was computed from the slope of the linear portion of the cumulative amount of drug permeated per square centimetre versus time plot. The permeability coefficient was determined by dividing the steady-state flux by the initial drug load, as shown in Equation (2), and the diffusion coefficient (D) was obtained using Equation (3):

(2) $$P_{c}\;=\;\frac{J_{\mathrm{SS}}}{C_{d}}$$

(3) $$D\;=\;{\left(\frac{\mathrm{Slope}}{2C_{d}}\right)}^{2}\times \pi$$

where *C*_d_ is the initial drug concentration in the donor compartment, and the slope is that of the cumulative amount of drug permeated versus the square root of time plot.

The enhancement ratio (ER) was calculated by dividing the *J*_ss_ of the optimised microemulsion system by that of the sildenafil suspension [4,19,20].

### 2.12. In Vitro Drug Metabolism

#### 2.12.1. Cell Culture Condition

The human liver cell line (HepG2; ATCC^®^ HB-8065™, Manassas, VA, USA) was used in this study. HepG2 cells were cultured in Dulbecco’s modified Eagle’s medium (Gibco^®^, Grand Island, NY, USA) containing 10% FBS (Gibco^®^, USA) and antibiotics (100 U/mL penicillin and 100 U/mL streptomycin, Gibco^®^, Grand Island, NY, USA) under 5% CO_2_ at 37 °C. After the HepG2 cells were grown for 4 days, the cell cultures were treated with sildenafil citrate and the sildenafil microemulsion-loaded hydrogel at various concentrations (7, 9, and 11 μg/mL) in complete media and incubated at 37 °C in 5% CO_2_ for 24 h. After incubation, the cells were washed once with phosphate-buffered saline (pH 7.4; Difco, Franklin Lakes, NJ, USA) to remove unbound drugs, treated with 1 mL of 0.1% sodium dodecyl sulphate (Sigma-Aldrich, St. Louis, MO, USA), and incubated at 37 °C for 2 min to lyse the cells. After incubation, 4 mL of complete media was added to the culture flasks, and the supernatant was kept in a −20 °C freezer until assay.

#### 2.12.2. Liquid-Liquid Extraction Procedure

Liquid-liquid phase extraction was used to extract the drug and its metabolites from the samples before further analysis. The supernatant samples were thawed to room temperature. The sample (1 mL) was added to 3 mL of the extraction mixture (ether (60%) and dichloromethane (40%)) and then vortexed for 3 min. The top organic layer, which contained the drug and internal standard, was transferred into a new tube. This extraction process was repeated to increase the content of the extracted drug. The extracted sample was evaporated to remove the organic solvents until dryness was reached with a stream of oxygen-free nitrogen at 40 °C. The residue was reconstituted with 1 mL of the HPLC mobile phase, and a 50 μL sample was injected into the HPLC system to determine the content of the drug and its metabolite in the extracted samples according to the method described in Section 2.8. Diazepam was used as an internal standard at a concentration of 1 μg/mL.

#### 2.12.3. Determination of Sildenafil and Its Metabolite Using Liquid Chromatography with Tandem Mass Spectrometry

The liquid chromatography with tandem mass spectrometry (LC-MS/MS) system consisted of a Shimadzu Prominence LC system (controller CBM-20A, pumps LC-30AD, and autosampler SIL-30AC; Shimadzu, Tokyo, Japan) with an ion trap time-of-flight mass spectrometer (Shimadzu, Tokyo, Japan). The column used for the separation was an Ascentis^®^ Express C18, 2.7 µm (50 × 3.0 mm; Supelco Analytical, Darmstadt, Germany). The LC mobile phases consisted of 50% water in 0.1% formic acid (A) and 50% acetonitrile in 0.1% formic acid (B) at a flow rate of 0.25 mL/min at room temperature. The ion spray voltage was 4.5 kV. Nitrogen was used as the nebulising gas at 1.50 L/min. The curved desolvation line (CDL) and heat block temperatures were set to 200 °C. The mass spectrometer was operated in the positive ion mode in the range of *m/z* 150–600 with the collision energy at 50%. Data analysis and acquisition were performed using ACD/Labs (Version S05S41).

### 2.13. Statistical Analysis

The results were statistically analysed using Student’s *t*-test (*p* < 0.05). All measurements were performed in triplicate, and the data are presented as the average values with the standard deviation. 

## 3. Results and Discussion

### 3.1. Microemulsion Systems

Development of a microemulsion containing the poorly water-soluble drug, sildenafil, requires ingredients that exhibit excellent drug solubilization. A previous study prepared sildenafil (base) microemulsions, and the results revealed that the solubility of sildenafil was considerably higher (by approximately 10 times) in oleic acid (75.5 ± 11.3 mg/mL) than that of sildenafil citrate in the present study. However, the solubility of sildenafil (base) in surfactants showed the opposite trend [21]. The water solubility of sildenafil and sildenafil citrate was reported to be 0.0 ± 0.0 mg/mL and 3.20 ± 0.11 to 4.1 ± 1.3 mg/mL, respectively [5,22]. Sildenafil citrate exhibits a higher water solubility compared to the sildenafil base; however, sildenafil citrate requires oil to improve its permeability through the skin. The higher solubility of sildenafil (base) in oil than sildenafil citrate in oil can be attributed to the polarity of the citrate salt and the low oil partition coefficient of the salt form. However, because the objective of this study was to load sildenafil citrate in microemulsion systems together with a hydrogel to develop a new formulation, sildenafil citrate was used.

The saturated solubility study of sildenafil citrate in various oils, surfactants, and co-surfactant is shown in Table 2. The highest solubility of sildenafil citrate was obtained with oleic acid (7.63 ± 0.48 mg/mL), followed by isopropyl myristate, while the lowest solubility was obtained with castor oil. The association between the weak basic drug sildenafil (pKa 8.7) and the acidic oleic acid could be responsible for the highest solubility in this oil. These results are similar to those of a previous report by Elshafeey et al. [5] and Jung et al. [22], although the solubility values are different. The experiments on the solubility of sildenafil citrate in different surfactants demonstrated the highest solubility in Tween 80 among all the surfactants tested, followed by Cremophore RH40. These results contrast with the previous report by Elnaggar et al. [23], which placed Cremophore RH40 higher than Tween 40 in terms of sildenafil citrate solubility. In the case of co-surfactants, PEG400 and propylene glycol exhibited the maximum solubility of sildenafil citrate (5.16 ± 0.22 and 4.10 ± 0.58 mg/mL, respectively). Considering the solubility of sildenafil citrate, isopropyl myristate and oleic acid should be the most appropriate oils for developing microemulsions as the solubility of sildenafil citrate in isopropyl myristate and oleic acid is 7.63 ± 0.48 and 4.21 ± 0.65 mg/mL, respectively. Thus, both oleic acid and isopropyl myristate are considered as suitable oil phases due to the high drug solubility and low risk of skin toxicity. Therefore, the surfactant and co-surfactant systems selected were Tween 80, PEG400, and propylene glycol. Oleic acid was used as the oil phase to construct the phase diagram for the microemulsion systems because it was a common absorption enhancer in transdermal excipients and possesses the suitable properties of surfactants and co-surfactants.

**Figure 2:**
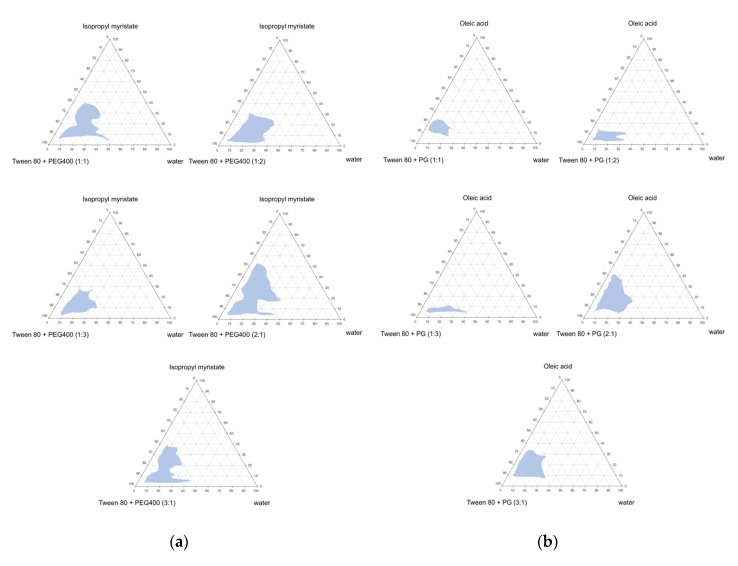
Pseudo-ternary phase diagrams of various ratios of co-surfactant (Tween 80/PEG400), oil (isopropyl myristate) and water (**a**) and co-surfactant (Tween 80/propylene glycol), oil (oleic acid) and water (**b**). The microemulsion regions of the ternary plots are indicated as light blue areas.

The pseudo-ternary phase diagrams of the microemulsions were constructed using isopropyl myristate or oleic acid as the oil phase. Tween 80 was used as a surfactant and PEG400 or propylene glycol was used as a co-surfactant at a surfactant-to-co-surfactant ratio of 1:1, 1:2, 1:3, 2:1, or 3:1. The pseudo-ternary phase diagrams obtained from these systems are displayed in Figure 2.

The construction of a pseudo-ternary phase diagrams helps in determining the concentration range of the components to check for the existence of microemulsions. Microemulsion systems are formed at room temperature. When water was added to the selected oil mixtures (a mixture of isopropyl myristate with Tween80/PEG400 or a mixture of oleic acid with Tween 80/propylene glycol) in all formulations, a continuous transition from water-in-oil systems (W/O) to oil-in-water (O/W) systems was observed, and a transparent, one-phase, and low-viscosity system was obtained. The O/W microemulsions formed are shown in a three-component triangular diagram. The transparent to translucent microemulsion region is shown in phase diagrams (Figure 2). No distinct phase inversion of the microemulsions was noted. The remaining region on the phase diagram represents the turbid and conventional emulsions based on visual observation. The incorporation of isopropyl myristate and Tween 80/PEG400 as the surfactant and co-surfactant in different ratios is shown in Figure 2a. When the maximum amount of Tween 80/PEG400-incorporated water was used in the oil-surfactant system, a microemulsion zone was formed. As the ratio of the surfactant-to-co-surfactant increased, the existence area of the microemulsion increased, reaching a maximum at 2:1. The addition of a surfactant and co-surfactant mixture in a 2:1 ratio increased the water incorporation to a maximum of 40% compared to 2% in the co-surfactant-free system.

The pseudo-ternary phase diagram of the oleic acid system obtained using Tween 80/propylene glycol as the surfactant and co-surfactant is shown in Figure 2b. The results were similar to those of the isopropyl myristate system. In addition, when the ratio of Tween 80 was low, the microemulsion area was small. This result indicates that the surfactant plays an important role in the microemulsion formation in this system.

### 3.2. Sildenafil Citrate Microemulsion-Loaded Hydrogel

Various microemulsion systems were selected from the phase diagram of Figure 2 for all ratios of surfactants and co-surfactants. The parameters of the microemulsions are shown in Table 3. All the microemulsions had small average droplet diameters ranging from 30 to 600 nm. The polydispersity index showed that all the microemulsions had a narrow size distribution. However, when sildenafil citrate was added to the microemulsion in large amounts (more than about 10%), a milky white liquid was obtained.

The composition of the microemulsion system used to prepare the microemulsion is shown in Table 3, and the solubility of sildenafil citrate in the microemulsion system was measured. In the isopropyl myristate system, the microemulsion contained 30% isopropyl myristate, 40% Tween 80, and 20% PEG400, and showed the lowest droplet size at 30 ± 6 nm and the highest sildenafil citrate solubility at 122 ± 28 mg/mL. Furthermore, this ratio contained a high content of Tween 80 (40–43%), which is used as an enhancer for transdermal delivery [24]. In the oleic acid system, the microemulsion contained 22% oleic acid, 43% Tween 80, and 22% propylene glycol gave the lowest droplet size of 320 ± 45 nm with the highest sildenafil citrate solubility of 128 ± 8 mg/mL. Notably, the solubility of sildenafil citrate in the microemulsion system was improved. Sildenafil citrate solubility reached approximately 4.21–7.63 mg/mL in isopropyl myristate, a 1.3–2.4-fold increase compared with its intrinsic solubility in water (3.20 ± 0.11 mg/mL) [5] and 38–40-fold increase compared to its solubility in the microemulsion of the isopropyl myristate or oleic acid systems (122 ± 28 and 128 ± 8 mg/mL), respectively. Thus, the composition ratios of 30:40:20:10 for isopropyl myristate:Tween 80:PEG400:water and 22:43:22:13 for oleic acid:Tween 80:propylene glycol:water were chosen for the isopropyl myristate-based microemulsion system and oleic acid-based microemulsion system, respectively. Notably, the solubility of sildenafil citrate in the oleic acid-based microemulsion was higher than that in the isopropyl myristate-based microemulsion system.

Based on these results, microemulsions containing 12% sildenafil citrate were prepared at a surfactant-to-co-surfactant ratio of 2:1. The microemulsions containing sildenafil citrate resulted in phase diagrams similar to those of the microemulsions without the drug. The detailed compositions of the three microemulsions are shown in Table 1, with the same composition as that shown in Table 3. All these formulations existed inside the area of microemulsion formation, thereby forming a clear microemulsion at the additive concentrations examined. Hydrogel-thickened microemulsions were formulated by mixing poloxamer 188 solution with the microemulsion.

**Figure 3:**
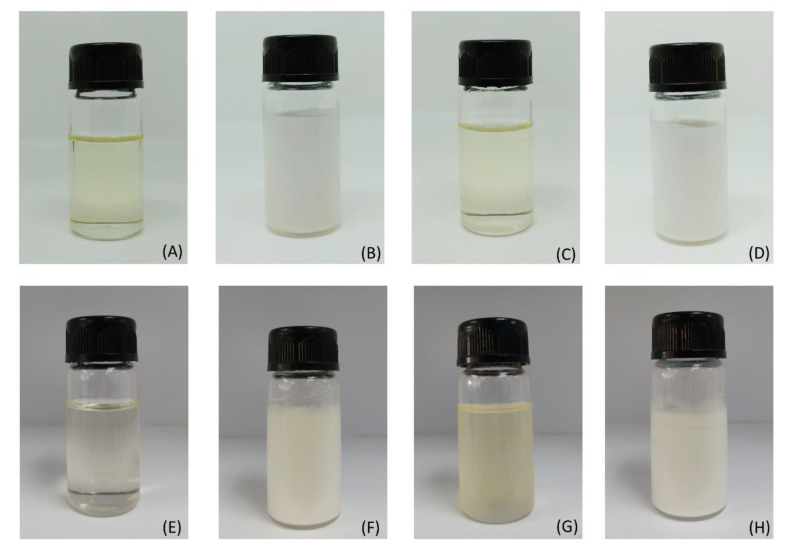
Photographs of the isopropyl myristate and oleic acid microemulsions system. The isopropyl myristate system is shown in the upper row and the oleic acid system is shown in the lower row; microemulsion without drug and hydrogel (**A**,**E**); sildenafil citrate dissolved in the microemulsion (**B**,**F**); microemulsion-loaded poloxamer 188 (**C**,**G**); and sildenafil citrate dissolved in the microemulsion and loaded with poloxamer 188 (**D**,**H**).

A photograph of the microemulsion of both the isopropyl myristate-based and oleic acid-based systems are shown in Figure 3. Microemulsions were clear, transparent, and had a low viscosity (Figure 3A,E). When 12% sildenafil citrate was added to the microemulsion, a milky white opaque microemulsion was obtained (Figure 3B,F). When poloxamer 188 was added to microemulsion in order to thicken the system without sildenafil citrate, the microemulsion-loaded hydrogel remained clear and transparent with the increasing formulation viscosity (Figure 3C,G). Finally, sildenafil citrate (12%) and poloxamer 188 were added to the microemulsion; consequently, highly viscous and milky white hydrogels were obtained (Figure 3D,H). The opacity of the sildenafil citrate microemulsion-loaded hydrogel occurred even when they were dissolved, but the presence of an off-white powder of sildenafil citrate caused a milky white emulsion and increased the particle size of the systems.

The physical properties of the sildenafil citrate microemulsion systems with and without poloxamer 188 are summarized in Table 4. The droplet size of the sildenafil citrate microemulsion-loaded hydrogel ranged from 461 ± 108 nm to 619 ± 68 nm, which was slightly higher than that of the microemulsion without poloxamer 188 (450 ± 120 nm to 525 ± 45 nm). This is caused by the swelling of the microemulsion in the hydrogel system. The pH of the sildenafil citrate microemulsion-loaded hydrogel of both formulations was 5.27 ± 0.04 and 4.67 ± 0.07 for the isopropyl myristate and oleic acid systems, respectively, compared to 5.14 ± 0.02 and 4.54 ± 0.03 for the formulation free of a gelling agent. These results indicate that poloxamer does not affect the pH of the formulation. At this pH range, the formulation the microemulsion system leads to less irritation when applied to the skin of the penis, especially the glans penis, which is sensitive to response.

The viscosity of the sildenafil citrate isopropyl myristate-based microemulsion was 4569 ± 79 mPa s, and that of the sildenafil citrate oleic acid-based microemulsion was 1256 ± 55 mPa s. Increasing viscosity was obtained with both the sildenafil citrate microemulsion systems when poloxamer 188 was added to the formulations. The viscosity of the isopropyl myristate-based and oleic acid-based microemulsion was 11,997 ± 1465 mPa s and 3231 ± 69 mPa s, respectively. The viscosity of the microemulsion was significantly increased by adding poloxamer 188 to the hydrogel.

Spreadability of the isopropyl myristate-based microemulsion-loaded hydrogel was lower than that of the oleic acid-based microemulsion-loaded hydrogel system because of the higher viscosity of the isopropyl myristate-based formulation. Spreadability is a particularly important parameter, as it shows the behaviour of the microemulsion-loaded hydrogels and the ease with which the formulation is applied to the glans penis.

### 3.3. FTIR Analysis

**Figure 4:**
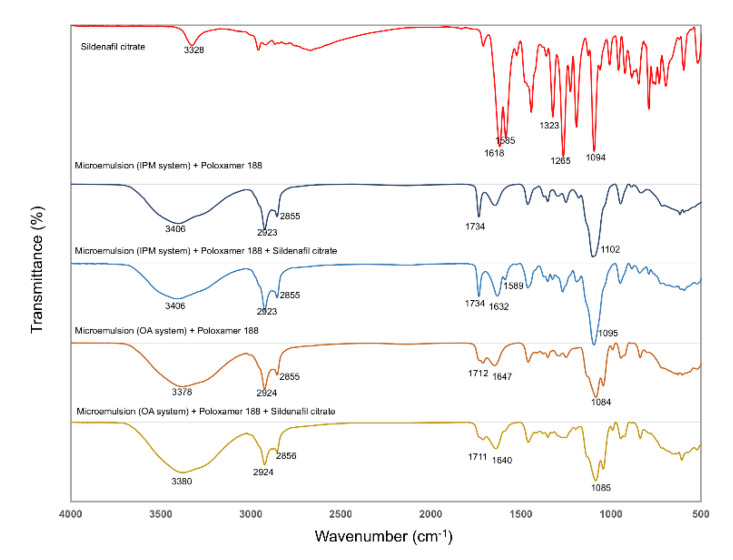
The FTIR spectra of sildenafil citrate, the microemulsion-loaded hydrogels, and the sildenafil microemulsion-loaded hydrogel over the region of 400–4000 cm^−1^.

The IR spectra in the frequency region from 400 to 4000 cm^−1^ for sildenafil citrate and for the microemulsion-loaded hydrogels with and without sildenafil citrate (with both the isopropyl myristate and oleic acid-based systems) are shown in Figure 4, and the interpretation is shown in Table 5. The asymmetric (~1255 cm^−1^) and symmetric (~1323 cm^−1^) SO_2_ stretching bands associated with the molecular vibration of the sulphone group are shown in Table 5, and are similar to that reported in previous studies [25,26]. After the sildenafil citrate dissolved in the microemulsion, both the asymmetric and symmetric SO_2_ stretching bands were absent. The IR spectra of the microemulsions of both the isopropyl myristate- and the oleic acid-based systems showed a broad and strong band in the range 3300–3500 cm^−1^ (owing to O–H stretching of the gel). The peaks centred around 2850–2950 cm^−1^ arise from the C–H stretching peaks of all samples. The bands at 1632 and 1640 cm^−1^ are the so-called N–H bend bands of amide in the sildenafil microemulsion of the isopropyl myristate and oleic acid-based systems, respectively. The band shift to higher wavenumbers of sildenafil citrate from 1618 cm^−1^ to 1632–1640 cm^−1^ led to the bending of the secondary amide in the cyclic amide, which nearly overlapped with the rich C=O stretching band of the microemulsion systems [27]. The band at 1589 cm^−1^ in the spectrum can be attributed to the symmetric stretching frequency of the COOH groups belonging to the citrate ion [25] appearing in the isopropyl myristate-based system, but not in the oleic acid-based system.

### 3.4. Rheological Analysis

In this study, poloxamer 188 was selected because it is commonly used in pharmaceutical preparations and is readily soluble in water [28]. Poloxamer 188 solution (10%) was added to the microemulsion system to prepare the microemulsion-based hydrogel after dissolving sildenafil citrate into the microemulsion system. However, after poloxamer 188 completely swelled in the microemulsion, it still exhibited a transparent and clear characteristic. We found that poloxamer 188 increased the viscosity of the microemulsion, maintained the microemulsion structure, and was a good matrix that swelled in the microemulsion system.

**Figure 5:**
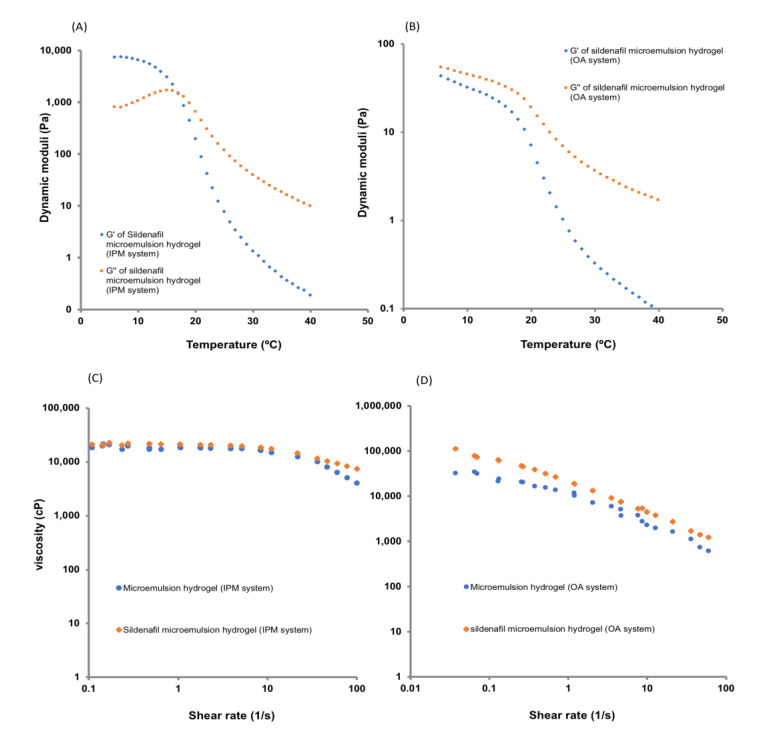
Temperature dependence of the dynamic moduli of isopropyl myristate system (**A**), and oleic acid system (**B**); viscosity curve of the sildenafil microemulsion-loaded hydrogels in different formulations of isopropyl myristate system (**C**), and oleic acid system (**D**).

The viscosity and elasticity of the hydrogel samples can be monitored using the parameters of elastic modulus (G′) and loss modulus (G″) [29]. The viscous liquid or elastic hydrogel shows that G′ is smaller than G″. Furthermore, a larger G′ reflects the inherent characteristics of the solid samples, and the phase transition from liquid to semisolid. The rheological properties of the microemulsion-loaded hydrogel with and without sildenafil citrate are shown in Figure 5. The sildenafil citrate microemulsion-loaded hydrogel showed a shear thinning system.

The temperature sweep plots of the hydrogels are shown in Figure 5. The behaviour of the two hydrogels was significantly different, indicating a difference in their structure. For the hydrogel prepared from the isopropyl myristate-based microemulsion system, throughout the temperature sweep, both during heating and cooling, G′ was higher than G″ at temperatures below 18 °C, and the values of both moduli were larger than those of the hydrogels prepared from the oleic acid-based microemulsion system.

The isopropyl myristate microemulsion hydrogel exhibited a thermo-reversible nature; the gel showed breaks near 18 °C and remained in the sol phase at higher temperatures. The hydrogel retained its sol form after cooling to a temperature of 18 °C. These results suggest that crosslinks are responsible for the gel breaks at higher temperatures for the hydrogel prepared at a neutral pH, and the hydrogel remains stable only at low to moderate temperatures.

### 3.5. Analytical Validation of Sildenafil Citrate in the Formulation

Analytical validation was performed to determine whether the analytical method was precise and accurate for determining sildenafil citrate in the microemulsion-loaded hydrogel system and the stability of the drug through the shelf-life. The specificity was investigated by observing any compound interference from the excipients present in the formulations. The results showed that none of the compounds in the isopropyl myristate- or oleic acid-based microemulsion system interfered with this analytical method (see Figure S1 in the supplementary files). Linearity was observed in the range of 0.020–40 mg/mL, which is suitable for sample preparation for analysis. The correlation coefficient (r^2^) was 1.000, with the equation y = 615.82x + 1.5235, where y is the peak area of sildenafil citrate in milli-absorbance unit (mAU) and x is the concentration of sildenafil citrate in mg/mL. The accuracy was determined by calculating the percentage recoveries for sildenafil citrate using the standard addition method at the levels of 80%, 100%, and 120%. The average percentage recovery was 97.18% ± 3.77%, indicating that this analytical method can be used to assay sildenafil citrate in the microemulsion system. The precision result for three days showed that the % labelled amount (LA) of sildenafil citrate was 99.50% ± 2.85% and the percentage relative standard deviation (RSD) or repeatability was 1.23% ± 0.43%. The intermediate precision was 2.7%.

### 3.6. Stability

The stability testing of the sildenafil citrate microemulsions of the two systems is shown in Table 6. No significant changes in appearance, pH, and viscosity were found when these preparations were stored at 30 °C and 75% RH for 6 months and in accelerated conditions at 40 °C and 75% RH, indicating that the hydrogel loaded with sildenafil citrate was physically stable. The assay content of sildenafil citrate in the microemulsion-loaded hydrogel was within the range of 90.0–110.0% LA of sildenafil in both formulations (the isopropyl myristate- and oleic acid-based systems). At 1 month in the oleic acid-based system, the sildenafil content was 112%, as the sample preparation was not homogeneous; however, at the next time point, the content still remained within the range. When the sildenafil citrate microemulsion-loaded hydrogel in the oleic acid-based system was accelerated for 1 month, phase separation occurred. These results may be attributed to the oxidative degradation of oleic acid, which is the most expected chemical instability of this system. In addition, phase separation may easily occur due to the low viscosity of the oleic acid-based microemulsion system.

### 3.7. In Vitro Skin Permeation Studies

**Figure 6:**
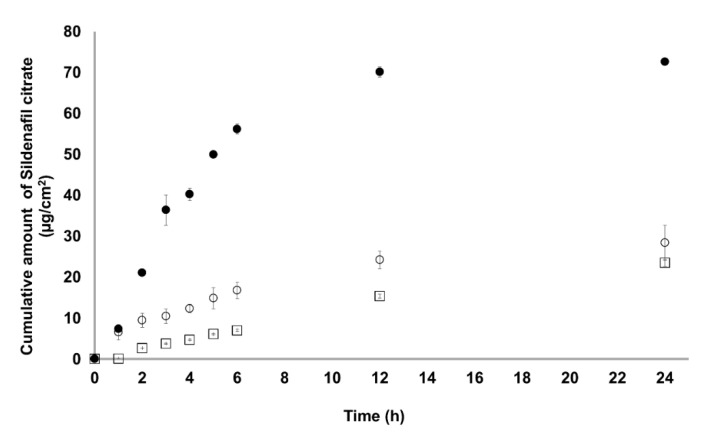
Transdermal permeation of the sildenafil citrate microemulsion-loaded hydrogel (● isopropyl myristate-based system; ○ oleic acid-based system) and sildenafil citrate suspension (⎕) across the Strat-M^®^ membrane in a citrate buffer solution at pH 5.8 (*n* = 6).

The permeation rates of the sildenafil citrate-loaded hydrogel from the microemulsion across the membrane from the microemulsion-loaded hydrogel are shown in Figure 6, and the skin permeation parameters are shown in Table 7. The isopropyl myristate-based microemulsion system showed the highest permeation rate (2.105 ± 0.003 μg/cm^2^/h), followed by sildenafil citrate suspension (1.070 ± 0.177 μg cm^−2^ h^−1^) and oleic acid-based microemulsion system (0.984 ± 0.244 μg/cm^2^/h). The isopropyl myristate-based microemulsion system showed the highest permeation rate of this drug. This may be attributed to the permeation-enhancing effect of isopropyl myristate, Tween 80, and PEG400, with a lower droplet size in the microemulsion. Although isopropyl myristate, oleic acid, and Tween 80 have also been frequently used as powerful permeation enhancers, isopropyl myristate acts as a more effective permeation enhancer for topical delivery in microemulsion systems [30].

The skin fluxes of sildenafil citrate from the isopropyl myristate-based microemulsion system were two times higher than those of the oleic acid-based microemulsion system and sildenafil citrate suspension. This result might be attributed to the considerably higher solubility and diffusion rates of sildenafil citrate from the microemulsions as a liquid medium than those from the sildenafil citrate solution without any enhancer. Statistical comparison of the flux throughout 24 h showed that the isopropyl myristate-based microemulsion system provided fluxes (*p* < 0.05) higher than those of the saturated solution of sildenafil citrate and oleic acid-based microemulsion system. The permeation profiles of the sildenafil citrate microemulsions followed zero-order release kinetics. A possible explanation is that the surfactant and co-surfactant may exist in each phase of the microemulsion; hence, the active ingredient can be partly solubilised in the external phase, and the depletion of the active ingredient in the external phase because of the permeation into the skin can be supplemented by releasing the active ingredient from the internal phase. Further, the zero-order release kinetics and sustained, controlled, and prolonged delivery of active ingredient were obtained. Oleic acid and propylene glycol as permeation enhancers had a strong permeation-enhancing effect and could enhance the solubility of sildenafil citrate in the skin. The partition coefficient could be increased owing to permeation enhancers. In addition, because of the small droplet diameters of the microemulsions, the likely mechanism may also be the permeation of sildenafil citrate directly from the droplets into the stratum corneum without microemulsion fusion to the stratum corneum and the subsequent permeation enhancement. The decrease in oleic acid release rate compared to that of the control can be attributed to the mechanism of fatty acids, such as partitioning into lipid bilayers, i.e., the stratum corneum, and the formation of lipophilic complexes with the drugs [31].

### 3.8. Cytotoxicity of the Sildenafil Citrate Microemulsion-Loaded Hydrogel to Human Skin Epithelial Cells (BJ Cells)

The percentage of human skin epithelial cell (BJ cell) viability was evaluated when it was incubated with the sildenafil citrate isopropyl myristate-based or oleic acid-based microemulsion-loaded hydrogel in the concentration range 1.95–1000 mg/mL for 24 h. This experiment was performed to ensure that the formulation was not harmful to the cells where it was applied. The percentage of cell viability are shown in Figure S2. The results revealed that both the sildenafil citrate microemulsion-loaded hydrogel systems did not induce cytotoxicity in BJ cells at all the tested concentrations.

### 3.9. Drug Metabolism

The accumulation of drugs after the pure sildenafil citrate suspension and sildenafil citrate microemulsion-loaded hydrogel (isopropyl myristate system) were incubated with HepG2 cells is shown in Figure S3 (see supplementary files). The sildenafil citrate microemulsion-loaded hydrogel could penetrate cells at a 6 times higher rate than that of the sildenafil suspension.

**Figure 7:**
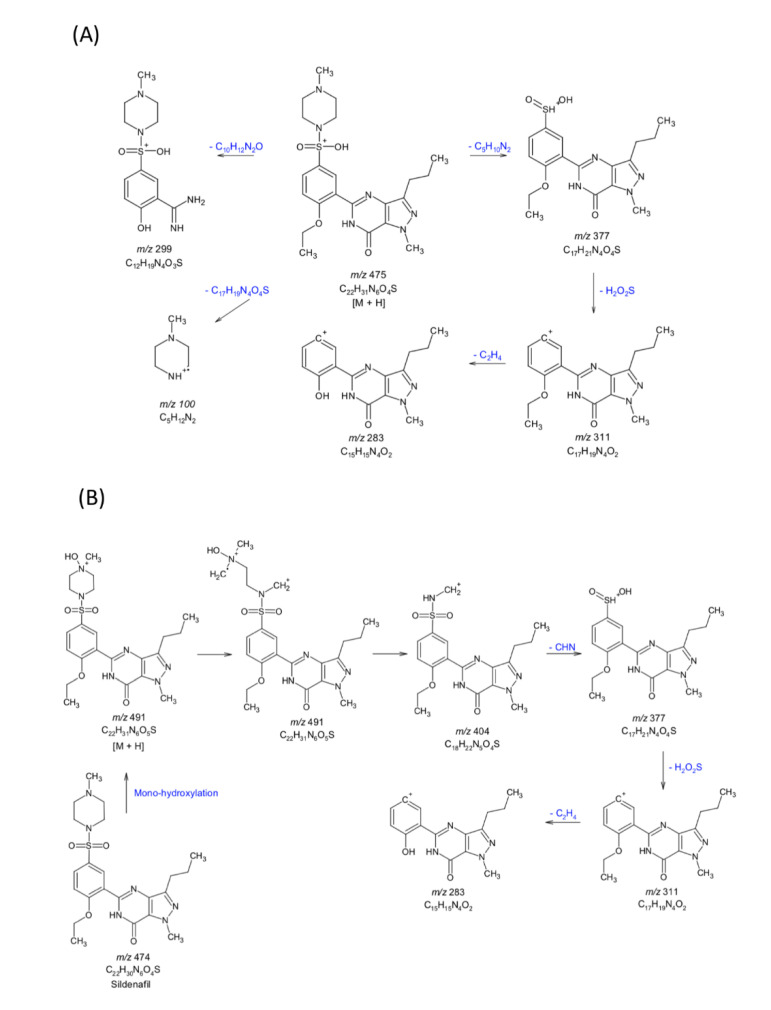
Proposed metabolic pathways of sildenafil citrate (**A**) and the sildenafil citrate microemulsion-loaded hydrogel (IPM system) (**B**) from the LC-MS/MS analysis.

The LC-MS/MS chromatogram of sildenafil citrate (pure drug) and the drug extracted from the supernatant of the HepG2 cells incubated with the sildenafil citrate microemulsion-loaded hydrogel or sildenafil aqueous suspension for 6 h is shown in Figure S4 (see supplementary files). Pure sildenafil citrate and sildenafil citrate extracted from the HepG2 cell supernatant incubated in suspension showed similar results. However, different mass results were obtained from the sildenafil citrate extracted from the HepG2 cell supernatant incubated with the sildenafil citrate microemulsion-loaded hydrogel. The proposed drug metabolism pathways of the sildenafil citrate aqueous suspension and sildenafil citrate microemulsion-loaded hydrogel are shown in Figure 7. The sildenafil citrate pure drug suspension induces the following metabolic pathways: piperazine *N*-demethylation; pyrazole *N*-demethylation *N*,*N*-deethylation (*m/z* 299, 283, 377, 311); and aliphatic hydroxylation [32]. This result contrasts with the sildenafil citrate microemulsion-loaded hydrogel showing mono-hydroxylation (*m/z* 491) of sildenafil citrate, which is a major metabolite of over 90% of the parent compound. The metabolism of the sildenafil citrate microemulsion-loaded hydrogel was concentration-dependent; consequently, when a high drug concentration was applied, the metabolism was saturated. In addition, the metabolite of the sildenafil formulation was decreased by approximately 50% compared to that of the sildenafil suspension.

## 4. Conclusions

In this study, a novel formulation of a sildenafil citrate microemulsion-loaded hydrogel was successfully developed for transdermal application and characterised in vitro. It was developed by checking the solubility of the drug in various oils, surfactants, and co-surfactants using pseudo-ternary phase diagrams, and was then optimized suitably for applying to the skin. The microemulsion composed of isopropyl myristate or oleic acid (oil) and a mixture of surfactant and co-surfactant (Tween 80/PEG400 or Tween 80/PG) was prepared, and poloxamer 188 was added to the microemulsion-loaded hydrogel. The membrane permeation rate of the microemulsion-loaded hydrogel was 1.97 times higher than that of the pure drug solution. According to the results of the characterisation, stability, and in vitro permeation studies, the most desirable formulation for the topical delivery of sildenafil citrate was considered to be the isopropyl myristate-based microemulsion system. Moreover, it did not result in cytotoxicity in the cell line studied. The metabolism of the sildenafil citrate microemulsion-loaded hydrogel was concentration-dependent and decreased compared to the sildenafil citrate aqueous suspension. Moreover, the metabolic pathways were different and decreased sildenafil metabolism from the hydroxylation pathway compared to the sildenafil citrate aqueous suspension.

## Figures and Tables

**Table 1:** Composition of the sildenafil citrate microemulsion-loaded hydrogel formulations.

Ingredients	Formulations and Amount (g)	
	Isopropyl Myristate-Based System	Oleic Acid-Based System
Sildenafil citrate	0.24	0.24
Isopropyl myristate	0.60	-
Oleic acid	-	0.44
Tween 80	0.80	0.87
Polyethylene glycol 400	0.40	-
Propylene glycol	-	0.43
Purified water	0.20	0.26
Poloxamer 188 (10% solution)	0.50	0.50
Total weight	2.74	2.74

**Table 2:** Saturated solubility of sildenafil citrate in different solvents at room temperature (27 °C ± 2 °C).

Component	Solvent	Saturated Solubility (mg/mL)
Oils	Oleic acid	7.63 ± 0.48
	Isopropyl myristate	4.21 ± 0.65
	Palm oil	3.65 ± 0.19
	Sesame oil	3.16 ± 0.73
	Olive oil	2.54 ± 0.22
	Castor oil	1.89 ± 0.62
Surfactants	Tween 80	12.14 ± 0.45
	Cremophore RH40	10.21 ± 0.32
	Tween 40	8.15 ± 0.13
	Tween 20	7.22 ± 0.43
Co-surfactants	PEG400	5.16 ± 0.22
	Propylene glycol	4.10 ± 0.58
	Transcutol	3.88 ± 0.15
Water	Distilled water	3.20 ± 0.11–4.1 ± 1.3 *

Solubility values are the mean ± SD, *n* = 3. * Solubility data of sildenafil in water from references [5,22].

**Table 3:** Droplet size and saturated solubilities of sildenafil citrate in different microemulsion systems at room temperature.

Composition of Microemulsion Systems (%)					Droplet Size (nm)	Solubility of Sildenafil Citrate (mg/mL)
Isopropyl Myristate	Tween 80/PEG 400	Oleic Acid	Tween 80/Propylene Glycol	Purified Water		
30	25:25 (1:1)	-	-	20	35 ± 10	72 ± 12
20	20:40 (1:2)	-	-	20	62 ± 12	58 ± 4
20	16:49 (1:3)	-	-	15	45 ± 8	82 ± 16
30	40:20 (2:1)	-	-	10	30 ± 6	122 ± 28
20	49:16 (3:1)	-	-	15	110 ± 20	108 ± 14
-	-	15	37.5:37.5 (1:1)	10	593 ± 126	108 ± 12
-	-	10	25:50 (1:2)	15	385 ± 81	103 ± 9
-	-	8	18:54 (1:3)	20	600 ± 125	95 ± 24
-	-	22	43:22 (2:1)	13	320 ± 45	128 ± 8
-	-	15	52.5:17.5 (3:1)	15	465 ± 114	112 ± 16

Droplet size and solubility values are the mean ± SD, *n* = 3.

**Table 4:** Physical properties of the microemulsion systems and sildenafil citrate microemulsion-loaded hydrogel formulations.

Test	Sildenafil Citrate Microemulsion		Sildenafil Citrate Microemulsion-Loaded Hydrogel	
	(IPM System)	(OA System)	(IPM System)	(OA System)
Appearance	White liquid	White liquid	White liquid	White liquid
Clarity	Not clear	Not clear	Not clear	Not clear
Droplet size (nm)	450 ± 120	525 ± 45	461 ± 108	619 ± 68
pH	5.14 ± 0.02	4.54 ± 0.03	5.27 ± 0.04	4.67 ± 0.07
Viscosity (mPa s)	4569 ± 76	1256 ± 55	11,997 ± 1465	3231 ± 69
Spreadability (g cm s^−1^)	12.28 ± 2.35	17.65 ± 1.23	16.44 ± 2.12	19.45 ± 1.62
Swelling index (%)	-	-	11.0 ± 1.1	14.5 ± 1.2

All values are the mean ± SD, *n* = 3–6. IPM: isopropyl myristate; OA: oleic acid.

**Table 5:** Interpretation of the functional group and wave number by FTIR analysis.

Sample	Frequency (cm^−1^)	Interpretation	Compound class
Sildenafil citrate	3328	N–H stretch	
	1618	N–H bend	
	1585	Carboxylic acid	(citrate)
	1265	SO_2_	(symmetric)
	1323	SO_2_	(asymmetric)
	1094	C-N	
Microemulsion (IPM system) + poloxamer 188	3406	OH	(H_2_O), poloxamer, PEG400
	2923, 2855	C–H alkane	
	1734	COO–R	
	1102	C–O	
Microemulsion (IPM system) + poloxamer 188 + Sildenafil citrate	3406	OH	
	2923, 2855	C–H alkane	
	1734	COO–R	
	1632	N–H bend	(sildenafil)
	1589	Carboxylic acid	(citrate)
Microemulsion (OA system) + poloxamer 188	3378	OH	(H_2_O), poloxamer, PG
	2924, 2855	C–H alkane	
	1712	COOH	(oleic acid)
	1647	C=C alkene	(oleic acid)
	1102	C–O	
Microemulsion (OA system) + poloxamer 188 + Sildenafil citrate	3380	OH	(H_2_O), poloxamer, PG
	2924, 2856	C–H alkane	
	1711	COOH	
	1640	N–H bend	(sildenafil)
	1085	C–O	

**Table 6:** Content of sildenafil citrate and stability of the sildenafil citrate microemulsion-loaded hydrogel at 30 °C and 75% relative humidity (RH) and 40 °C and 75% RH for 6 months.

Formulation	Test	Storage Conditions						
		Initial	30 °C/75% RH			40 °C/75% RH		
			1 Month	3 Month	6 Month	1 Month	3 Month	6 Month
Sildenafil microemulsion (IPM system)	Appearance	White colour liquid	White colour liquid	White colour liquid	White colour liquid	White colour liquid	White colour liquid	White colour liquid
	Assay sildenafil (%)	104.5 ± 0.001	107.4 ± 0.28	106.5 ± 0.11	105.4 ± 0.13	99.1 ± 0.19	102.3 ± 0.25	101.1 ± 0.34
	pH	5.27 ± 0.04	4.91 ± 0.06	5.02 ± 0.00	5.07 ± 0.02	4.79 ± 0.06	4.89 ± 0.03	4.97 ± 0.02
	Viscosity (m Pa s)	11,997 ± 1465	7909 ± 1988	10,025 ± 1222	9923 ± 2456	10,035 ± 465	12,214 ± 1165	11,234 ± 2086
Sildenafil microemulsion (OA system)	Appearance	White colour liquid	White colour liquid	White colour liquid	Phase separation	Phase separation	Phase separation	Phase separation
	Assay sildenafil (%)	97.0 ± 0.002	112.8 ± 1.26	105.2 ± 2.18	108.6 ± 4.52	112.7 ± 3.58	NA	NA
	pH	4.67 ± 0.07	4.73 ± 0.03	4.66 ± 0.02	4.55 ± 0.02	4.45 ± 0.04	4.65 ± 0.03	4.58 ± 0.02
	Viscosity (m Pa s)	3231 ± 69	3372 ± 189	3344 ± 256	3312 ± 175	3605 ± 70	NA	NA

All values are the mean ± SD, *n* = 6–10. NA = Not applicable.

**Table 7:** Skin permeation parameters of the sildenafil citrate formulations through a Strat-M membrane in vitro after 24 h.

Parameter	Sildenafil Microemulsion (IPM System)	Sildenafil Microemulsion (OA System)	Sildenafil Citrate Suspension
Cumulative amount at 24 h (μg/cm^2^)	60.54 ± 0.62	28.42 ± 4.31	23.47 ± 0.86
Steady-stat flux (*J*_ss_) (μg cm^−2^ h^−1^)	2.11 ± 0.00 *	0.98 ± 0.24	1.07 ± 0.18
Permeability coefficient (P_c_) (cm h^−1^)	10.76 × 10^−5^ *	5.03 × 10^−5^	10.47 × 10^−5^
Diffusion coefficient (D) (cm^2^ h^−1^)	1.07 × 10^−6^ *	5.62 × 10^−6^	7.59 × 10^−6^
Enhancement ratio (ER)	1.97	0.92	-

Note: * significant variance (*p*-value < 0.05); all values are the mean ± SD, *n* = 6.

## Supplementary Materials

The following are available online at https://www.mdpi.com/1999-4923/12/11/1055/s1, Figure S1: The specificity validation for HPLC analytical method for the quantitation of sildenafil citrate in the microemulsion-loaded hydrogel system. (A) Sildenafil citrate in isopropyl myristate microemulsion-loaded hydrogel system, (B) sildenafil citrate in oleic acid microemulsion-loaded hydrogel system, (C) standard sildenafil citrate spike in the isopropyl myristate microemulsion-loaded hydrogel system, (D) standard sildenafil citrate spike in the oleic acid microemulsion-loaded hydrogel system, (E) placebo in the isopropyl myristate microemulsion-loaded hydrogel system, (F) placebo in the oleic acid microemulsion-loaded hydrogel system, Figure S2 Cell viability in HepG2 cells using the MTT assay after being induced with sildenafil citrate microemulsion-loaded hydrogel for 24 h. Data are shown as mean ± SD of triplicate assays. Figure S3: Amount of sildenafil citrate accumulation in the cell after incubation for 6 h, Figure S4: LC-MS/MS chromatogram of (A) sildenafil citrate, (B) drug extraction from cells incubated in sildenafil citrate aqueous solution for 6 h, (C) drug extraction from cells incubated in the sildenafil citrate microemulsion-loaded hydrogel (IPM system) for 6 h.

## Abbreviations

PDE5	phosphodiesterase type 5
EDHF	endothelium derived hyperpolarizing factor
PGI_2_	prostacyclin
cAMP	cyclic adenosine monophosphate
cGMP	cyclic guanosine monophosphate
NO	nitric oxide
eNOS	endothelial nitric oxide synthase
VEGF	vascular endothelial growth factor
PKA	protein kinase A
PKG	protein kinase G
PEG400	polyethylene glycol 400
HPLC	high-performance liquid chromatography
FTIR	Fourier transform infrared spectroscopy
FBS	foetal bovine serum
MTT	3-(4,5-dimethylthiazolyl-2-yl)-diphenyl-tetrazolium bromide
RH	relative humidity
LC-MS/MS	liquid chromatography with tandem mass spectrometry
