# Supplementary material for: Development of a Sildenafil Citrate Microemulsion-Loaded Hydrogel as a Potential System for Drug Delivery to the Penis and Its Cellular Metabolic Mechanism
Source: Pharmaceutics. 2020 Nov 4;12(11):1055. doi: 10.3390/pharmaceutics12111055 (PMC7694282; doi:10.3390/pharmaceutics12111055)
Supplement: Supplementary file 1 [file pharmaceutics-12-01055-s001.pdf]

pharmaceutics

Development of Sildenafil Citrate Microemulsion-Loaded Hydrogel as Potential Systems for Drug Delivery to the Penis and Its Cellular Metabolic Mechanism

Apichart Atipairin, Charisopon Chunhachaichana, Titpawan Nakpheng, Narumon Changsan, Teerapol Srichana and Somchai Sawatdee

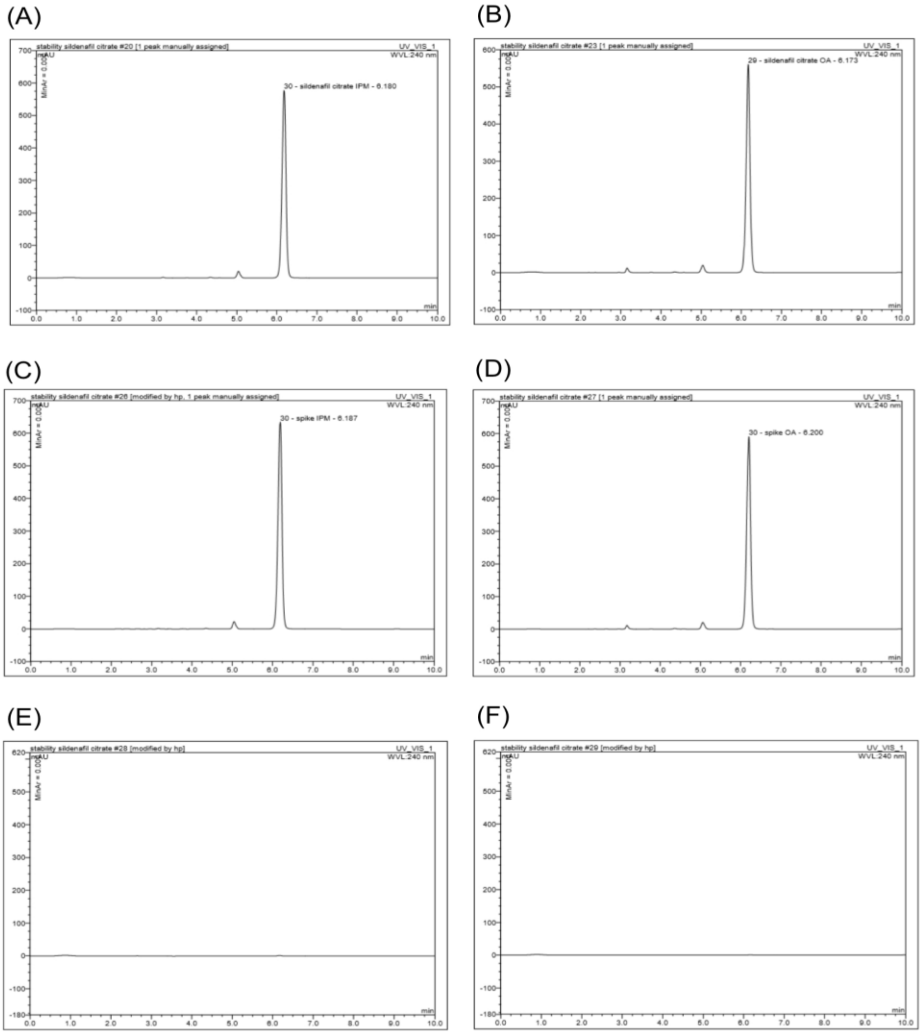

Figure S1. The specificity validation for HPLC analytical method for the quantitation of sildenafil citrate in the microemulsion-loaded hydrogel system. (**A**) Sildenafil citrate in isopropyl myristate microemulsion-loaded hydrogel system, (**B**) sildenafil citrate in oleic acid microemulsion-loaded hydrogel system, (**C**) standard sildenafil citrate spike in the isopropyl myristate microemulsion-loaded hydrogel system, (**D**) standard sildenafil citrate spike in the oleic acid microemulsion-loaded hydrogel system, (**E**) placebo in the isopropyl myristate microemulsion-loaded hydrogel system, (**F**) placebo in the oleic acid microemulsion-loaded hydrogel system.

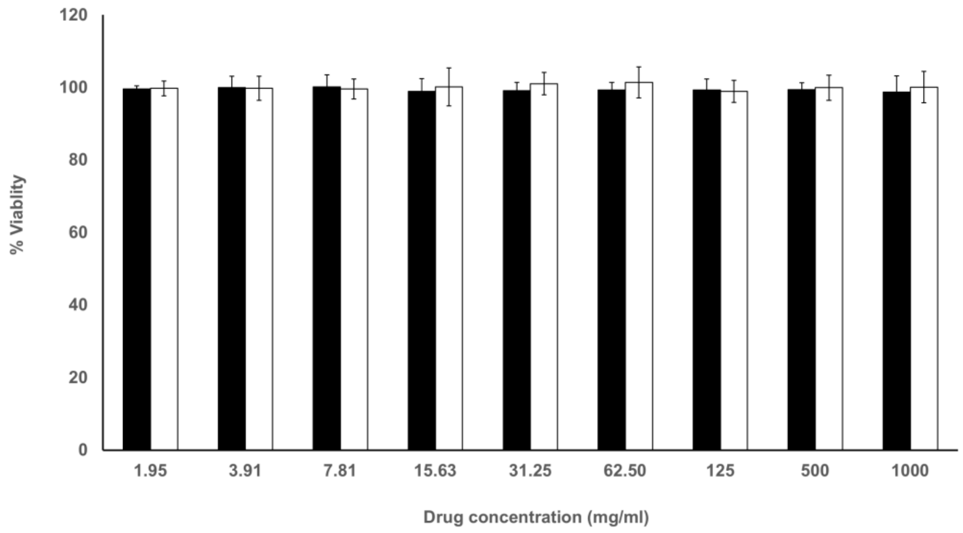

Figure S2. Cell viability in HepG2 cells using the MTT assay after being induced with sildenafil citrate microemulsion-loaded hydrogel for 24 h. Data are shown as mean ± SD of triplicate assays.

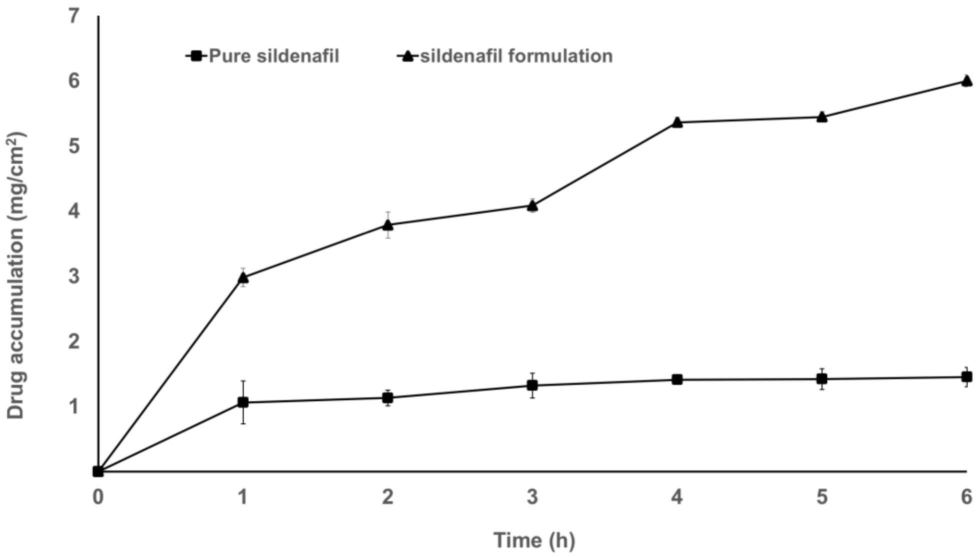

Figure S3. Amount of sildenafil citrate accumulation in the cell after incubation for 6 h.

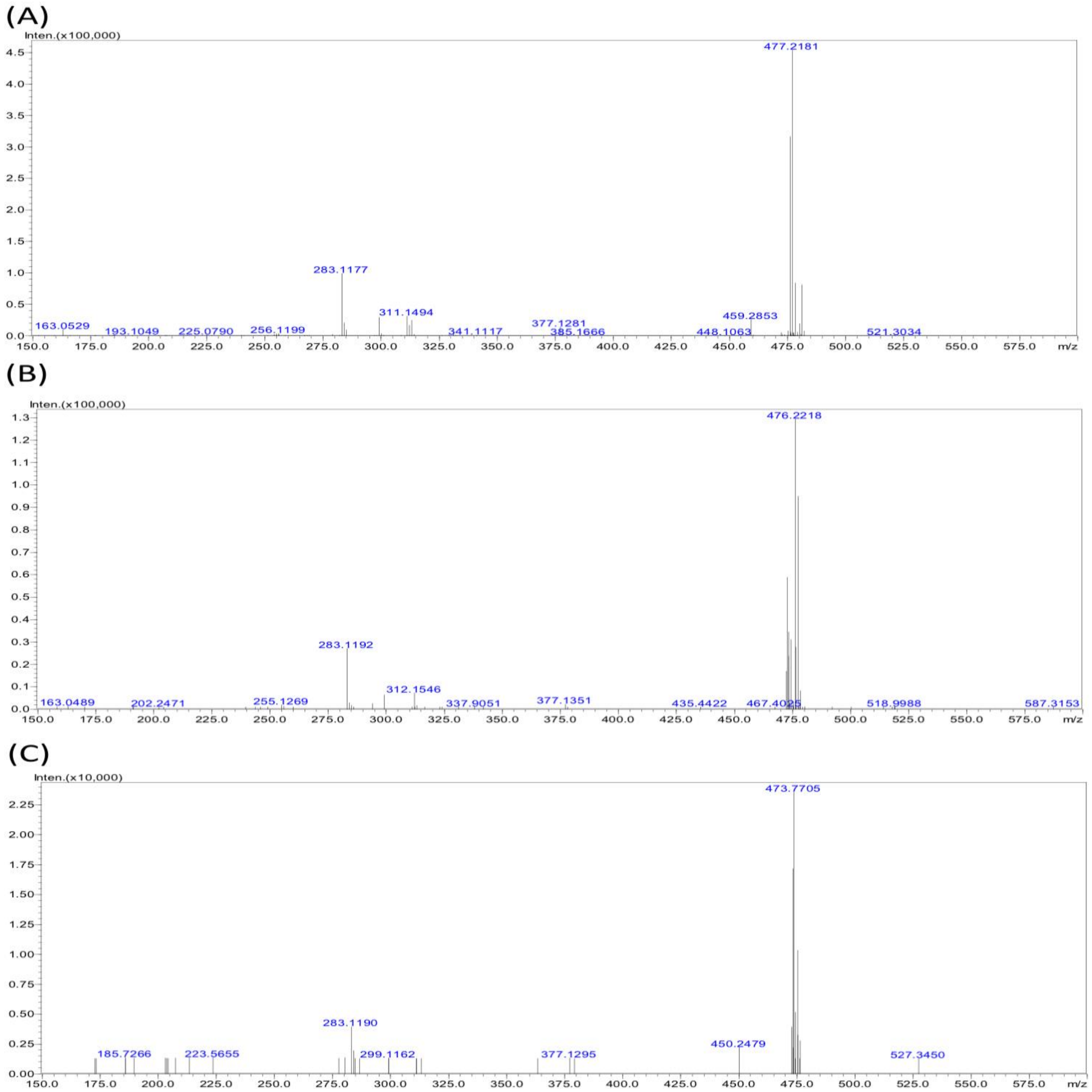

Figure S4. LC-MS/MS chromatogram of (**A**) sildenafil citrate, (**B**) drug extraction from cells incubated in sildenafil citrate aqueous solution for 6 h, (**C**) drug extraction from cells incubated in the sildenafil citrate microemulsion-loaded hydrogel (IPM system) for 6 h.
